# Altered serum protein levels in frontotemporal dementia and amyotrophic lateral sclerosis indicate calcium and immunity dysregulation

**DOI:** 10.1038/s41598-020-70687-7

**Published:** 2020-08-13

**Authors:** Jared S. Katzeff, Fiona Bright, Kitty Lo, Jillian J. Kril, Angela Connolly, Ben Crossett, Lars M. Ittner, Michael Kassiou, Clement T. Loy, John R. Hodges, Olivier Piguet, Matthew C. Kiernan, Glenda M. Halliday, Woojin Scott Kim

**Affiliations:** 1 Brain and Mind Centre, The University of Sydney, Camperdown, Sydney, NSW 2050 Australia; 2 School of Medical Sciences, The University of Sydney, Sydney, NSW Australia; 3 School of Mathematics and Statistics, The University of Sydney, Sydney, NSW Australia; 4 Sydney Mass Spectrometry, The University of Sydney, Sydney, NSW Australia; 5 Dementia Research Centre and Department of Biomedical Sciences, Macquarie University, Sydney, NSW Australia; 6 School of Chemistry, The University of Sydney, Sydney, NSW Australia; 7 The Garvan Institute of Medical Research, Sydney, NSW Australia; 8 School of Psychology, The University of Sydney, Sydney, NSW Australia; 9 Neuroscience Research Australia, Sydney, NSW Australia; 10 Institute of Clinical Neurosciences, Royal Prince Alfred Hospital, Sydney, NSW Australia; 11 School of Medical Sciences, University of New South Wales, Sydney, NSW Australia

**Keywords:** Biochemistry, Neuroscience

## Abstract

Frontotemporal dementia (FTD) and amyotrophic lateral sclerosis (ALS) are neurodegenerative diseases that are considered to be on the same disease spectrum because of overlapping genetic, pathological and clinical traits. Changes in serum proteins in FTD and ALS are poorly understood, and currently no definitive biomarkers exist for diagnosing or monitoring disease progression for either disease. Here we applied quantitative discovery proteomics to analyze protein changes in FTD (N = 72) and ALS (N = 28) patient serum compared to controls (N = 22). Twenty three proteins were significantly altered in FTD compared to controls (increased—APOL1, C3, CTSH, EIF5A, MYH2, S100A8, SUSD5, WDR1; decreased—C1S, C7, CILP2, COMP, CRTAC1, EFEMP1, FBLN1, GSN, HSPG2, IGHV1, ITIH2, PROS1, SHBG, UMOD, VASN) and 14 proteins were significantly altered in ALS compared to controls (increased—APOL1, CKM, CTSH, IGHG1, IGKC, MYH2; decreased—C7, COMP, CRTAC1, EFEMP1, FBLN1, GSN, HSPG2, SHBG). There was substantial overlap in the proteins that were altered in FTD and ALS. These results were validated using western blotting. Gene ontology tools were used to assess functional pathways potentially dysregulated in the two diseases, and calcium ion binding and innate immunity pathways were altered in both diseases. When put together, these results suggest significant overlap in pathophysiological peripheral changes in FTD and ALS. This study represents the first proteomics side-by-side comparison of serum changes in FTD and ALS, providing new insights into under-recognized perturbed pathways and an avenue for biomarker development for FTD and ALS.

## Introduction

Frontotemporal dementia (FTD) is a neurodegenerative disease and a common form of younger-onset dementia, of which the most common clinical syndrome is the behavioral variant FTD (bvFTD)^1^. Amyotrophic lateral sclerosis (ALS) is the most rapidly fatal motor neurodegenerative disease, with typical progression from symptom onset to death in 2–3 years^2,3^. FTD and ALS are considered to be on the same disease spectrum because of overlapping genetic, pathological and clinical traits^4^. The most common gene abnormality in both FTD and ALS is an expanded hexanucleotide repeat sequence in the *C9ORF72* gene^5–7^. Pathologically, protein aggregates of TAR-DNA binding protein-43 (TDP-43), the microtubule associated protein tau or, less often, fused in sarcoma (FUS) are present in FTD and/or ALS brain.

Currently, there is a lack of sensitive and specific biomarkers for diagnosis and monitoring disease progression for FTD and ALS, which has hindered the capacity to develop therapies for the two diseases. Major pathological proteins, including TDP-43 and tau, have not provided accurate peripheral biomarkers (cerebrospinal fluid (CSF) or plasma/serum) for either disease^8–10^. As a result, other protein markers have been explored, with neurofilament-light chain (NfL) attracting considerable interest. NfL levels are elevated in both CSF and serum in both FTD and ALS^11,12^. Research into developing biomarkers specific to FTD and ALS is ongoing.

Proteomics is a technique for global quantification of protein abundance and is increasingly used to identify changes in protein levels in numerous diseases^13^. Proteomics technology has been recently applied to a number of neurodegenerative diseases for the purpose of biomarker development^14^. Only few proteomics studies have been carried out on ALS plasma^15–17^. However, to date no work has been reported on the proteomics of FTD serum/plasma nor any side-by-side comparisons of FTD and ALS proteins in serum/plasma. Here, we used proteomics based on mass spectrometry to analyze serum proteins in FTD and ALS serum compared to controls. The primary aim was to identify altered peripheral proteins in FTD and ALS that could be exploited to develop biomarkers for these diseases. The secondary aim was to uncover and understand FTD and ALS pathophysiology associated with any protein changes.

## Materials and methods

### Patient blood serum

Individuals diagnosed (male/female) with sporadic bvFTD (47/25), sporadic ALS (21/7) and healthy controls (9/13) were recruited from FRONTIER, the frontotemporal dementia clinical research group now at the University of Sydney Brain and Mind Centre, from the ForeFront FTD and motor neuron disease clinic at the University of Sydney Brain and Mind Centre, and from a panel of healthy study volunteers^18^ with no neurological or psychiatric disorders, notably no evidence of cognitive impairment. Two blood samples, taken 12 months apart (i.e. Year-1 and Year-2), were analyzed; in total, bvFTD (144 samples), ALS (56 samples) and controls (44 samples). The mean age at Year-1 was 61.5, 52.7 and 69.8 years respectively. The study was approved by the University of New South Wales (approval number: HC12573) and the University of Sydney (approval numbers: 2012/160, 2014/539, 2017/928) human research ethics committees. All methods were carried out in accordance with the relevant guidelines and regulations. Blood samples were obtained following written informed consent from the participant and/or primary carer. All patients underwent a neurological examination, a comprehensive cognitive assessment and structural brain MRI, and met current consensus diagnostic criteria for bvFTD^19^, ALS^20^ or no neurological disease. Blood samples (9 mL) were collected in tubes (BD Vacutainer SST II Advance Tube #367958), and serum prepared by centrifugation at 3,500 rpm for 10 min at 4 °C, which was then aliquoted and stored at − 80 °C until use.

### Protein depletion

The ‘top 14’ high abundant proteins were depleted from the samples using a 4.6 mm × 100 mm Multiple Affinity Removal System column (MARS, Agilent, Santa Clara, CA, USA) based on the depletion method^21^ and following the manufacturer’s instructions. Briefly, 40 µl of serum was diluted with 120 µl buffer A, passed through a 0.22 µm filter and centrifuged at 16,000*g*. The supernatant was injected into a MARS column and the flow through collected. The column was washed with buffer B, which elutes the bound proteins, before re-equilibrating with buffer A prior to the next sample. The collected fractions were buffer exchanged into 100 mM TEAB.

### Mass spectrometry

The buffer exchanged flow through from the depletion step was mixed with an equal volume of 8 M urea, and the proteins were reduced and alkylated then digested with trypsin. The peptides were purified using Oasis hydrophilic–hydrophobic-balanced (HLB) plus short cartridges (Waters Corp., Milford, MA, USA) and resuspended in 30 µl water, the peptide concentration assess using a QUBIT (Invitrogen, Carlsbad, CA, USA), and the pH adjusted to pH 8.0. The concentration was adjusted to 10 µg/30 µl with 100 mM TEAB and labelled with 10plex Tandem Mass Tags (TMT, Thermo Fisher Scientific, Waltham, MA, USA), following the manufacturer’s instructions. The TMT-labelled peptides were purified using a HLB column and fractionated by hydrophilic interaction liquid chromatography (HILIC) in off-line mode using an in-house packed TSK-Amide 80 HILIC column with PEEK filter and an Agilent 1200 chromatography system (Agilent Technologies, Santa Clara, CA, USA). Each of the ten HILIC fractions were dried down and then resuspended in MS loading buffer (3% (v/v) acetonitrile/0.1% (v/v) formic acid) and analyzed by nano-capillary liquid chromatography–tandem mass spectrometry (LC–MS–MS) using a Dionex Ultimate 3000 HPLC system (Thermo Fisher Scientific, Waltham, MA, USA) coupled to an in-house fritless nano 75 μm × 30 cm column packed with ReproSil Pur 120 C18 stationary phase (1.9 μm, Dr, Maisch GmbH, Germany). Separated compounds were analyzed with an Orbitrap Fusion Tribrid Mass Spectrometer (Thermo Fisher Scientific, Waltham, MA, USA). A synchronous precursor selection MS3 method^22^ was used for data collection. Proteome Discoverer 2.2 (Thermo Fisher Scientific, Waltham, MA, USA) was used to analyze the MS data as the runs progressed and any TMT plex with less than 500 protein groups was re-run. The raw mass spectrometry data was first processed using MaxQuant^23^. The variable modification included oxidation for methionine and protein N-terminal acetylation. A single fix modification of carbamidomethyl cysteine was included. Global parameters included a 1% false discovery rate.

### Gene ontology analysis

Two gene ontology software programs, Bioprofiling^24^ (https://www.bioprofiling.de, 16 Dec 2019) and STRING^25^ v11 (16 Dec 2019), were used to interpret and predict function or pathway on a set of proteins identified by the proteomics analysis. The 23 proteins that were significantly altered in FTD (APOL1, C1S, C3, C7, CILP2, COMP, CRTAC1, CTSH, EFEMP1, EIF5A, FBLN1, GSN, HSPG2, IGHV1, ITIH2, MYH2, PROS1, S100A8, SHBG, SUSD5, UMOD, VASN and WDR1) and the 14 proteins that were significantly altered in ALS (APOL1, C7, COMP, CKM, CRTAC1, CTSH, EFEMP1, FBLN1, GSN, HSPG2, IGHG1, IGKC, SHBG, MYH2) were entered separately into each of the programs following their instructions.

### Western blotting

Equal volumes of serum from randomly chosen samples were heated with sample buffer (3.2% SDS, 32% glycerol, 0.16% bromophenol blue, 100 mM Tris–HCl, pH 6.8, 8% 2-mercaptoethanol). They were then electrophoresed on Criterion Stain-free 4–20% SDS-PAGE gels (Bio-Rad) and transferred onto nitrocellulose membranes at 100 V for 30 min. The membranes were blocked with PBS containing 5% nonfat dry milk and probed separately with each of the antibodies: anti-CKM (mouse monoclonal, 1:1,000, Santa Cruz, sc-365046), anti-COMP (mouse monoclonal, 1:1,000, Santa Cruz, sc374660), anti-EFEMP1 (mouse monoclonal, 1:1,000, Santa Cruz, sc-33722, 1:1,000), anti-FBLN1 (mouse monoclonal, 1:1,000, Santa Cruz, sc25281), anti-GSN (mouse monoclonal, 1:1,000, Abcam, ab11081), anti-PROS1 (rabbit monoclonal, 1:1,000, Santa Cruz, sc-52720). The membranes were washed three times in PBS containing 0.1% Tween 20 and incubated with horseradish peroxidase-conjugated secondary antibodies for 2 h at room temperature. Signals were detected using enhanced chemiluminescence and Gel Doc System (Bio-Rad). The blots were stripped and probed for the housekeeper protein transferrin. The signal intensity was quantified using Image Lab (Bio-Rad) and NIH ImageJ software (v1.45s).

### Statistical analysis

In total, 855 proteins were detected across the entire experiment, with the percentage of missing proteins ranging from 27.1 to 51.9% across the different MS runs. Protein peak intensities were first log2 transformed, then any missing values were imputed using the k nearest neighbor algorithm (*impute.knn* function from the *impute* package in R). Following imputation, protein intensities were normalized across batches using the RUV-III (Removing Unwanted Variation-III) algorithm^26^. In the experimental design, samples were replicated across different runs to enable us to utilize RUV-III, which uses negative controls and replicates to remove systematic errors of unknown origin. All 855 proteins were used as negative controls. Default parameters from the *RUVIII* function were used. After normalization, any proteins that were originally missing were removed, and samples with replicates averaged. Then we fitted linear models using the R/Bioconductor software package limma^27^. A design matrix which included age and sex as covariates was used and tested for significance of disease status; neither age nor sex had any effect on protein levels. To test for differences across years, the dependent variable was set to the difference in protein values between the two years. The Benjamini–Hochberg method was used to control for multiple testing, and proteins with an adjusted p-value < 0.05 were considered to be statistically significant. For the western blotting data, statistical analysis was performed using SPSS Statistics software (IBM, Chicago, Illinois). Multivariate analysis (general linear model), and age and sex as covariates were used, and statistical significance set at *p* < 0.05.

## Results

### Proteomics analysis of bvFTD and ALS serum

Here we undertook a comprehensive analysis of bvFTD and ALS serum proteins using proteomics based on the advanced liquid chromatography-tandem mass spectrometry (LC–MS–MS) technology. In total 144 bvFTD, 56 ALS and 44 control samples were analyzed; two samples per individual (i.e. Year-1 and Year-2; 12-month apart) for all analysis. We used the protein depletion method in which 96% of 14 high-abundant proteins (e.g. albumin, IgG) were removed^21^. This strategy allows for the identification of less abundant proteins with a greater accuracy. Our aim was to identify peripheral proteins that are altered in bvFTD and ALS compared to controls, and to interpret and understand the pathophysiology associated with the protein changes observed.

**Figure 1:**
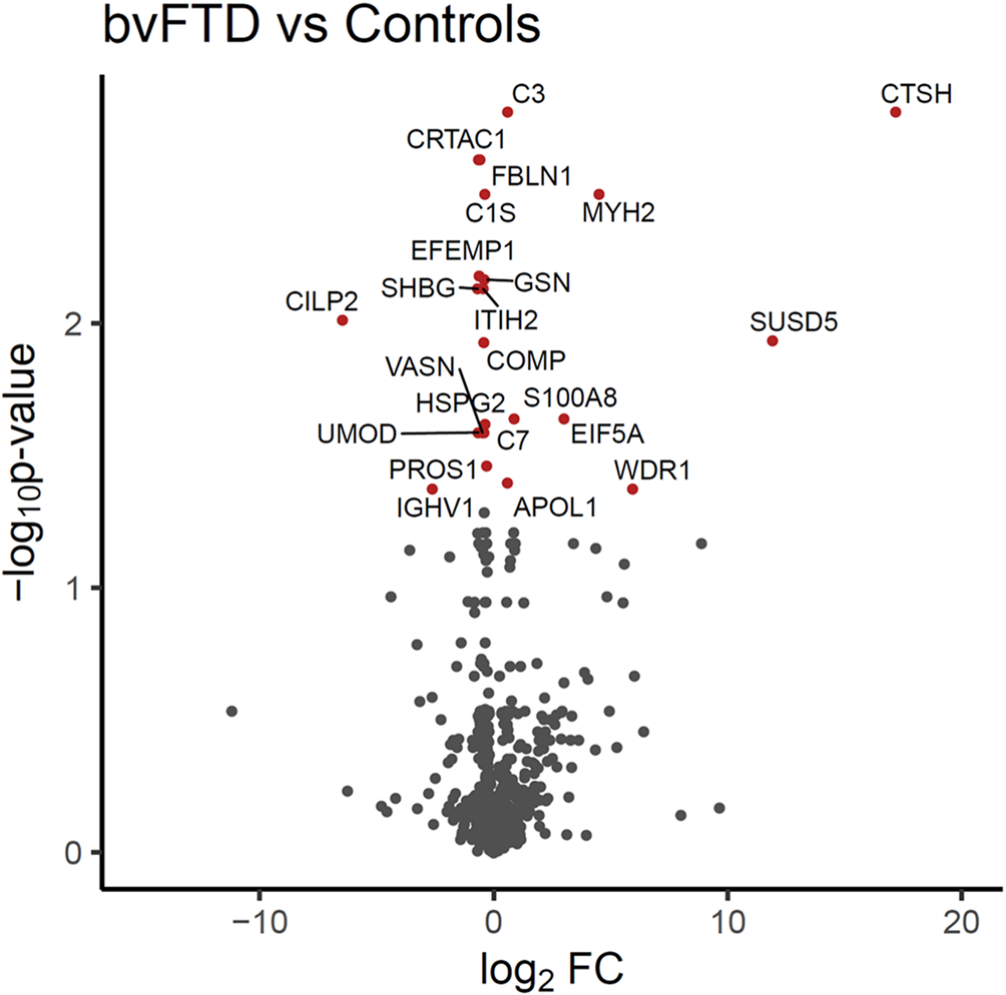
Volcano plot representation of bvFTD versus controls proteomics data. The significantly altered proteins in bvFTD (N = 72) compared to controls (N = 22) are shown as red circles. Age and gender were included as covariates, Benjamini–Hochberg method was used to correct for multiple testing, and proteins with an adjusted *P* < 0.05 to be statistically significant; *FC* fold change.

**Figure 2:**
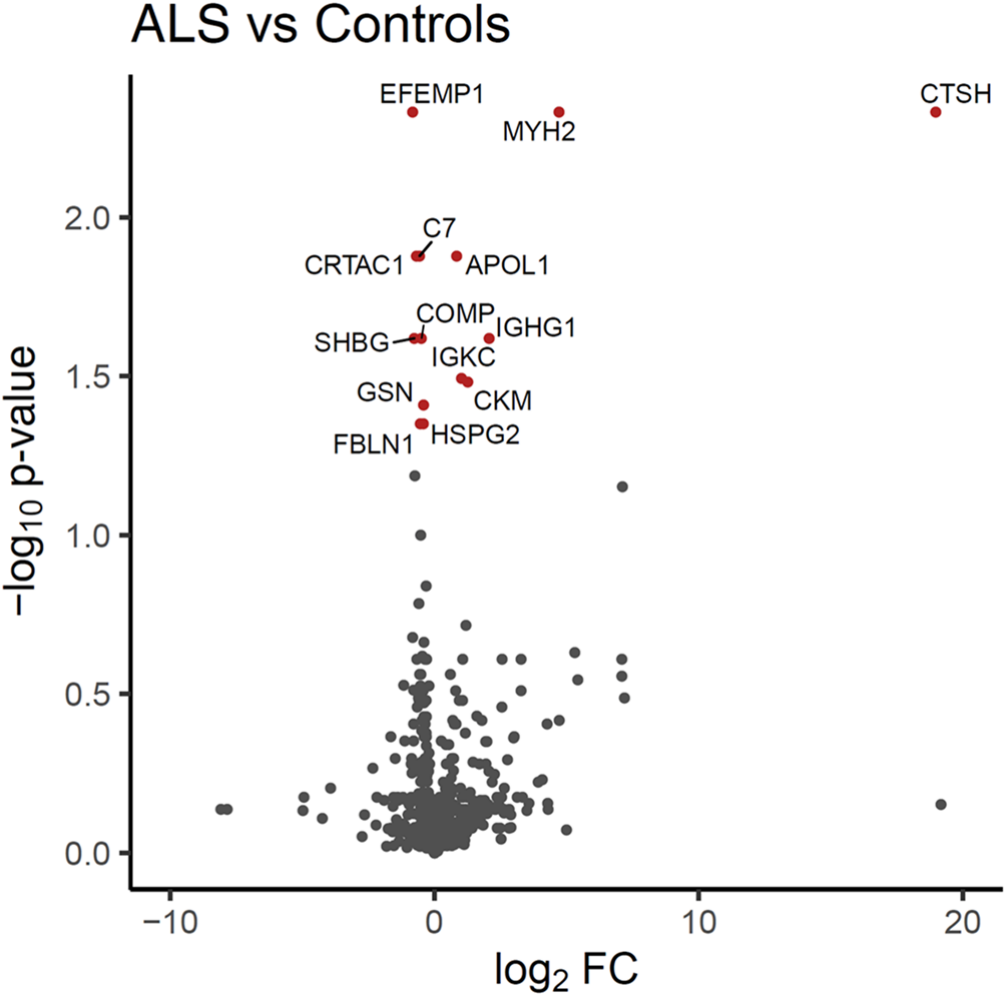
Volcano plot representation of ALS versus controls proteomics data. The significantly altered proteins in ALS (N = 28) compared to controls (N = 22) are shown as red circles. Age and gender were included as covariates, Benjamini–Hochberg method was used to correct for multiple testing, and proteins with an adjusted *P* < 0.05 to be statistically significant; *FC* fold change.

**Figure 3:**
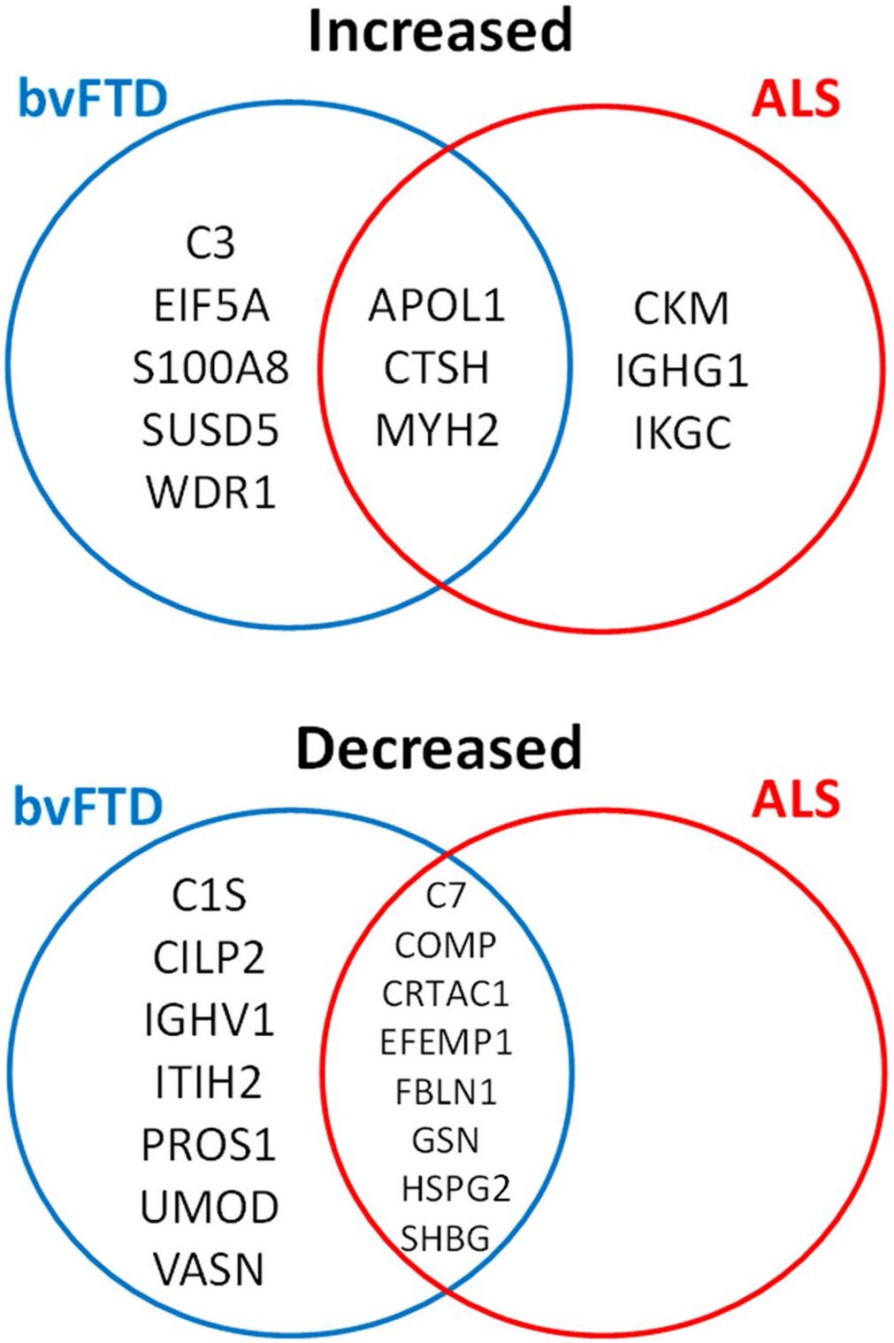
Overlap of proteins that were significantly altered in bvFTD and ALS serum. The blue circle represents those proteins that were significantly altered in bvFTD and red circle represents those proteins that were significantly altered in ALS. Three proteins were increased in both bvFTD and ALS. Eight proteins were decreased in both bvFTD and ALS.

Firstly, each disease was compared with controls, independent of year and covarying for age and sex. In bvFTD, 23 proteins were significantly altered (Fig. 1). Eight were increased—APOL1, C3, CTSH, EIF5A, MYH2, S100A8, SUSD5, WDR1—and 15 were decreased—C1S, C7, CILP2, COMP, CRTAC1, EFEMP1, FBLN1, GSN, HSPG2, IGHV1, ITIH2, PROS1, SHBG, UMOD, VASN (Table 1). In ALS, 14 proteins were significantly altered (Fig. 2) with 6 proteins increased—APOL1, CKM, CTSH, IGHG1, IGKC, MYH2—and 8 proteins decreased—C7, COMP, CRTAC1, EFEMP1, FBLN1, GSN, HSPG2, SHBG (Table 2). Interestingly, there was a substantial overlap in the proteins that were altered in the two diseases with 11 of the proteins altered in both diseases, i.e. APOL1, CTSH and MYH2 were upregulated, and C7, COMP, CRTAC1, EFEMP, FBLN1, GSN, HSPG2 and SHBG downregulated (Fig. 3).

**Table 1:** Proteins that were significantly altered in bvFTD compared to controls.

Protein	Symbol	Uniprot code	LogFC	P-value
**Increased**				
Complement C3	C3	P01024	0.59	0.0016
Cathepsin H	CTSH	P09668	17.19	0.0016
Myosin-2	MYH2	Q9UKX2	4.50	0.0033
Sushi domain-containing protein 5	SUSD5	O60279	11.93	0.0117
Eukaryotic translation initiation factor 5A	EIF5A	P63241	3.01	0.0230
Protein S100A-8	S100A8	P05109	0.87	0.0230
Apolipoprotein L1	APOL1	O14791	0.59	0.0401
WD repeat-containing protein 1	WDR1	O75083	5.94	0.0422
**Decreased**				
Cartilage acidic protein 1	CRTAC1	Q9NQ79	0.64	0.0024
Fibulin-1	FBLN1	P23142	0.59	0.0024
Complement C1S	C1S	P09871	0.38	0.0033
EGF-containing fibulin-like extracellular matrix protein 1	EFEMP1	Q12805	0.62	0.0067
Gelsolin	GSN	P06396	0.40	0.0069
Sex hormone-binding globulin	SHBG	P04278	0.69	0.0074
Inter-alpha-trypsin inhibitor heavy chain H2	ITIH2	P19823	0.45	0.0074
Cartilage intermediate layer protein 2	CILP2	Q8IUL8	6.47	0.0099
Cartilage oligomeric matrix protein	COMP	P49747	0.42	0.0118
Perlecan	HSPG2	P98160	0.37	0.0242
Uromodulin	UMOD	P07911	0.66	0.0259
Complement C7	C7	P10643	0.41	0.0259
Vasorin	VASN	Q6EMK4	0.48	0.0259
Vitamin K-dependent Protein S	PROS1	P07225	0.29	0.0345
Immunoglobulin heavy variable 1–2	IGHV1	A0A0G2JMI3	2.63	0.0422

**Table 2:** Proteins that were significantly altered in ALS compared to controls.

Protein	Symbol	Uniprot Code	LogFC	P-value
**Increased**				
Myosin-2	MYH2	Q9UKX2	4.72	0.0047
Cathepsin-H	CTSH	P09668	18.99	0.0047
Apolipoprotein L1	APOL1	O14791	0.84	0.0133
Immunoglobulin heavy constant gamma 1	IGHG1	P01857	2.07	0.0240
Immunoglobulin kappa constant	IKGC	P01834	1.02	0.0322
Creatine kinase M-type	CKM	P06732	1.26	0.0322
**Decreased**				
EGF-containing fibulin-like extracellular matrix protein 1	EFEMP1	Q12805	0.82	0.0047
Complement C7	C7	P10643	0.56	0.0133
Cartilage acidic protein 1	CRTAC1	Q9NQ79	0.68	0.0133
Cartilage oligomeric matrix protein	COMP	P49747	0.49	0.0240
Sex hormone-binding globulin	SHBG	P04278	0.76	0.0240
Gelsolin	GSN	P06396	0.41	0.0389
Fibulin-1	FBLN1	P23142	0.54	0.0447
Perlecan	HSPG2	P98160	0.42	0.0447

**Figure 4:**
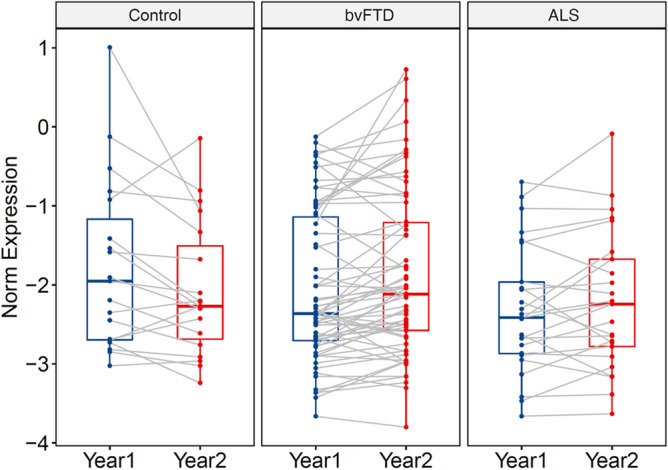
Changes in ENO1 abundance in a 12-month period in control, bvFTD and ALS serum. Blue dots represent Year-1 samples and red dots represents Year-2 samples; each individual is connected by a grey line. No significant change was observed for all three groups. Thick lines represent mean and boundary lines represent SE.

Secondly, Year-1 sample with Year-2 sample were compared within each group. There were no proteins that were significantly altered between the two time points for all three groups. The protein that was closest to significance was ENO1. ENO1 levels were non-significantly increased in bvFTD (*P* = 0.08), and unchanged in ALS and controls (Fig. 4).

### Validation of protein changes in bvFTD and ALS

**Figure 5:**
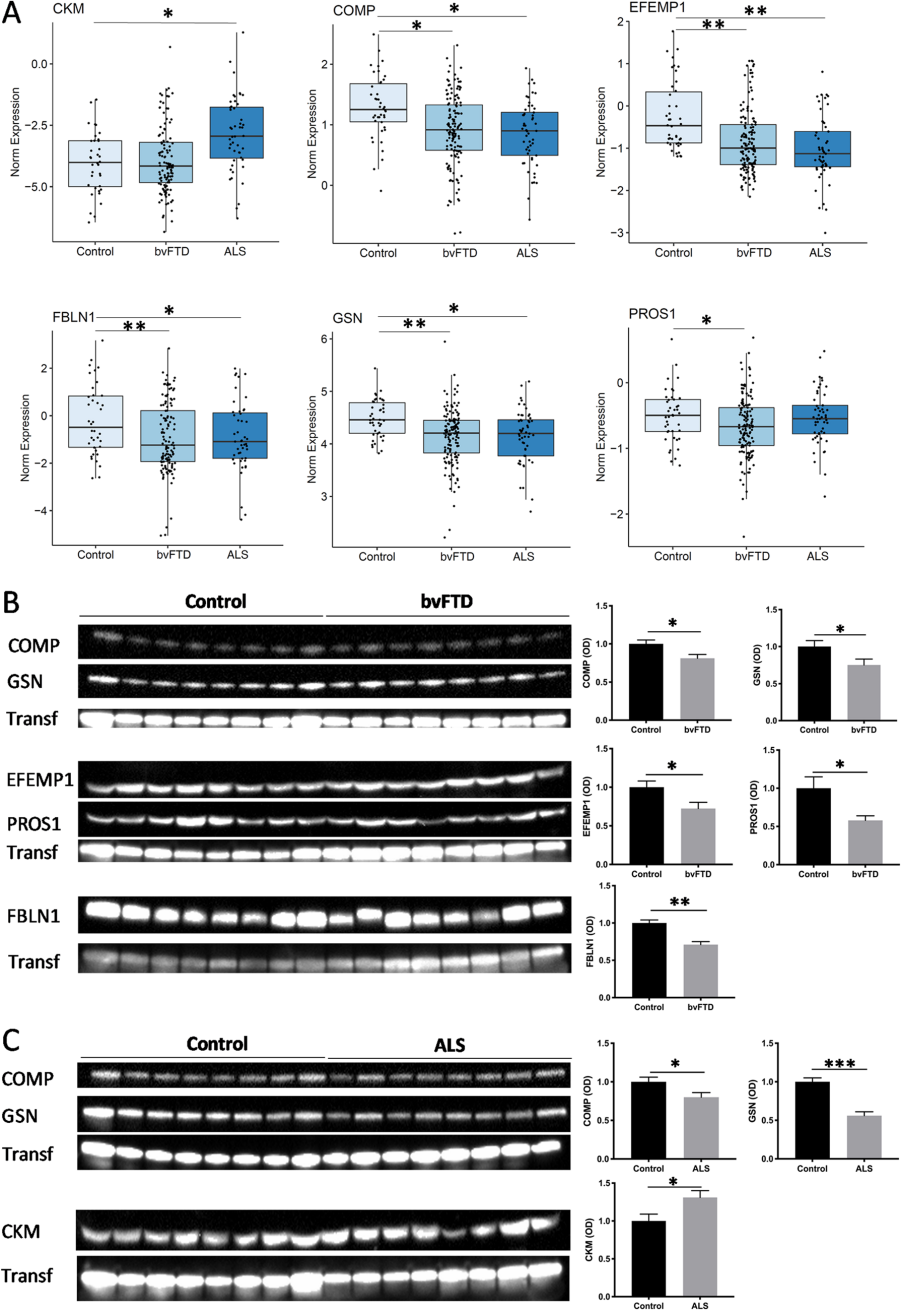
Validation of proteomics data by western blotting. (**A**) Proteomics data of individual proteins in bvFTD, ALS and controls. (**B**) Western blotting of individual proteins in bvFTD compared to controls normalized to the housekeeper protein transferrin (Transf), and optical density (OD) measurements of the bands. (**C**) Western blotting of individual proteins in ALS compared to controls normalized to the housekeeper protein transferrin (Transf), and optical density (OD) measurements of the bands. Data represent mean and SE as error bars, *P < 0.05, **P < 0.01, ***P < 0.001. The blots have been cropped from full-size blots as shown in the Supplementary Fig. 1.

The LC–MS–MS proteomics data was validated using western blotting. Proteins were selected for validation based on the availability of antibodies and suitability/sensitivity of protein detection in human serum by this method. Gene ontology results (see below) were also taken into account when selecting proteins for validation. Based on the gene ontology results, calcium ion binding was the prominent predicted function. The proteins selected for validation were therefore—COMP, EFEMP1, FBLN1, GSN and PROS (changed in bvFTD) and CKM, COMP, and GSN (changed in ALS). The same serum samples used for the proteomics analysis were electrophoresed on SDS-PAGE gels and probed with specific antibodies. All proteins tested were significantly altered in bvFTD and ALS compared to controls, supporting the proteomics data (Fig. 5).

### Assessing pathophysiological changes in bvFTD and ALS

**Figure 6:**
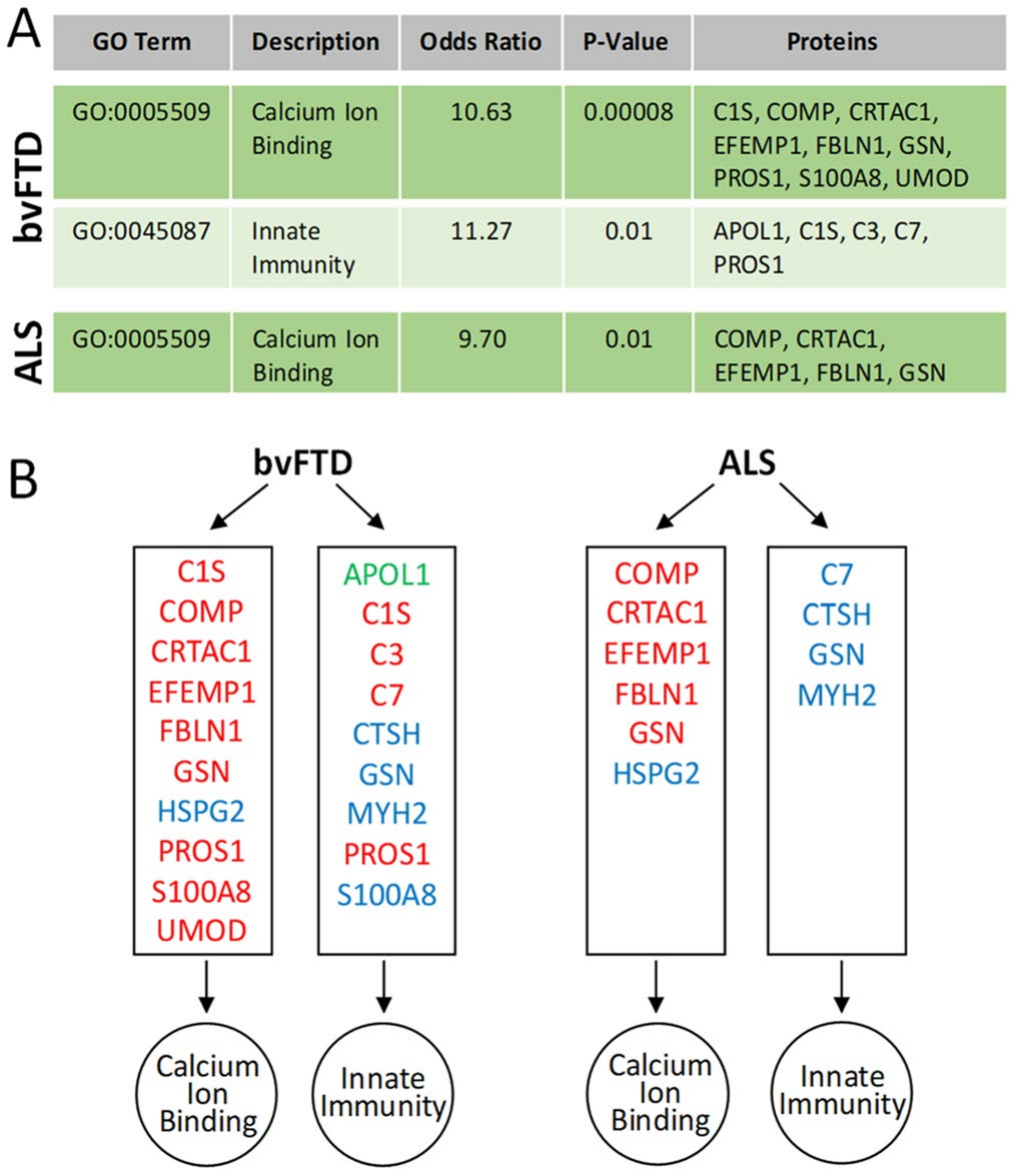
Gene ontology analysis of protein changes in bvFTD and ALS. (**A**) Potential pathways dysregulated in bvFTD and ALS as identified by Bioprofiling. (**B**) Proteins related to calcium ion binding and innate immunity. Red: identified by both Bioprofiling and STRING; green: bioprofiling only; blue: STRING only.

To understand the pathophysiology associated with the peripheral protein changes in bvFTD and ALS serum, two gene ontology software programs, bioprofiling and STRING, were used; gene ontology is a technique for interpreting and predicting functions or pathways based on changes in a set of genes or proteins. Firstly, we assessed the 23 proteins (APOL1, C1S, C3, C7, CILP2, COMP, CRTAC1, CTSH, EFEMP1, EIF5A, FBLN1, GSN, HSPG2, IGHV1, ITIH2, MYH2, PROS1, S100A8, SHBG, SUSD5, UMOD, VASN and WDR1) that were significantly altered in bvFTD by Bioprofiling. The prominent function/pathways generated were “calcium ion binding” pathway with 9 protein hits and “innate immunity” pathway with 5 protein hits (Fig. 6A), with the innate immunity pathway having the most clustering of upregulated proteins. The same two pathways (calcium ion binding and innate immunity) were identified using STRING with 10 and 8 protein hits respectively (Fig. 6B). Interestingly, all of the proteins (except S100A8) allocated to the two pathways using STRING were downregulated. In addition, four of the proteins (C1S, GSN, PROS1, S100A8) (Fig. 6B) overlapped in both pathways, suggesting a potential link between calcium ion binding and innate immunity in bvFTD.

Secondly, we assessed the 14 proteins (APOL1, C7, COMP, CKM, CRTAC1, CTSH, EFEMP1, FBLN1, GSN, HSPG2, IGHG1, IGKC, SHBG, MYH2) that were significantly altered in ALS by Bioprofiling and STRING. Similar to the protein changes in bvFTD serum, the prominent pathway generated by both programs was calcium ion binding with 5 and 6 protein hits respectively (Fig. 6B). There were also 4 protein hits in the innate immunity pathway identified using STRING (Fig. 6B).

## Discussion

Proteomics analysis of FTD and ALS has been conducted primarily in CSF with limited reproducibility across studies^16,28–31^. To date, no studies have been reported for FTD using serum or plasma. To address these shortfalls in knowledge, a comprehensive analysis of protein changes in bvFTD and ALS serum was performed using quantitative discovery proteomics. Twenty-three proteins were significantly altered in bvFTD and 14 proteins were significantly altered in ALS compared to controls, with 11 of these proteins altered in both diseases, supporting overlap in pathophysiological changes between FTD and ALS. Assessment of the altered proteins using two gene ontology programs established that the dysfunction primarily related to calcium ion binding pathways.

Calcium is recognized as critical to neurons given its multifunctional involvement in membrane excitability, signal transduction, neurotransmitter release, synaptic plasticity, cell cycle regulation and axon growth^32–34^. Existing evidence supports a link between calcium dysregulation and neurodegenerative processes in both FTD and ALS^35,36^. The drug Riluzole currently used to treat ALS works via inhibiting calcium signaling^37^. In a functional network study of an independent cohort of sporadic FTD patients, calcium/cAMP homeostasis and energetic metabolism impairments were identified as primary causes of the loss of neuroprotection and neural cell damage in FTD^36^. The authors suggested that calcium homeostasis in addition to DNA damage and oxidative stress could be among major molecular underpinnings for a significant proportion of unexplained FTD etiology^36^. In separate studies, calcium dysregulation was reported to contribute to neurodegeneration in iPSC-derived neurons from FTD patients^38^ and ALS patients^39^. In *C9ORF72* iPSC-derived motor neurons from ALS and FTD patients, decreased cell survival correlated with calcium homeostasis, suggesting a novel pathogenic link between *C9ORF72*, dysregulated calcium signaling and altered proteostasis^40^. Furthermore, in mutant models of TDP-43 pathology, increased calcium was shown to drive TDP-43 mediated neuronal toxicity^41^.

Collectively, these studies indicate that calcium dysregulation may be a critical component of neurodegenerative pathogenesis in both FTD and ALS. Given the current study shows dysregulation of multiple serum proteins associated with calcium binding pathways in both bvFTD and ALS, it may be necessary to monitor calcium homeostasis in living FTD, ALS and cognitively normal individuals to distinguish normal calcium homeostasis from pathological conditions. Of the calcium related proteins identified in our study, GSN has the strongest established relationship with neurodegeneration. GSN is primarily an actin binding protein, however it has other roles, including in neurotransmission, synaptic plasticity, cytoskeleton remodeling, apoptosis and inflammation^42,43^. Elevated calcium concentration causes GSN conformational changes that facilitate its binding to actin filaments^44^. Plasma GSN levels are decreased in ALS patients^15^, corroborating our results.

PROS1 is another protein involved in calcium ion binding. It has a well-established role in blood anticoagulation and is dependent on calcium binding for structural stability^45^. PROS1 is also an agonist for the TAM family of receptor tyrosine kinases, which are regulators of immunity. Given PROS1 is primarily produced by microglia in the brain, this suggests that it may play a role in neuroinflammation^46^. COMP is an extracellular matrix glycoprotein that contains calcium ion binding domains. Currently, little is known whether COMP plays a role in the human brain. FBLN1 and EFEMP1 are part of the fibulin family of extracellular matrix glycoproteins with numerous calcium binding domains^47,48^. FBLN1 is abundantly expressed in the human brain. It has been shown to bind to the amyloid precursor protein and modulate its activity^47^. EFEMP1 is thought to have anti-angiogenic properties^49^. CKM was upregulated only in ALS serum in the current study. CKM is a creatine kinase involved in energy consumption and, interestingly, elevated serum creatine is linked to muscle damage^50^. CKM has not been previously studied in the context of ALS. This protein is potentially a good candidate for follow-up studies, given ALS causes muscle weakness. Although the upregulation of ENO1 (enolase enzyme) in bvFTD serum did not reach significance over a one-year period, it will be useful to test this marker across longer disease progression. Enolase levels were shown to increase in AD CSF compared to controls, and these increases correlated with amyloid-β, total-tau and phosphorylated-tau levels^51,52^.

Whilst further investigation is required to understand the precise mechanisms and involvement of calcium dysregulation in neurodegeneration, serum calcium binding proteins, such as those validated in the current study, could be explored as potential indicators for neuronal vulnerability to disease^53,54^. The utility for such serum biomarkers to measure and detect calcium dysregulation in vivo in human subjects may facilitate tracking of specific at-risk neuronal populations in key neural networks within the CNS, that could allow for the development of neuroprotective approaches in FTD and ALS^53–55^.

Another prominent function/pathway generated by the gene ontology studies was innate immunity for both bvFTD and ALS. Innate immune-mediated mechanisms and chronic neuroinflammation are recognized as pathological hallmarks of neurodegenerative diseases, involved in both their development and progression^56,57^. In both FTD and ALS several key lines of evidence implicate innate immunity and neuroinflammation in the pathogenesis of disease^58,59^. Specifically, increased microglial activation and astrogliosis in disease affected brain regions^60,61^, altered expression of pro- and anti-inflammatory factors in blood and CSF^29,62^ and the most substantial evidence to date; the association between immune-related gene variants and susceptibility to FTD and ALS as recently reviewed in detail elsewhere^58,63^. Particularly in FTD, genome wide association studies consistently identify an association between the HLA locus (immune system) and FTD, and suggest a critical role for microglial and inflammation-associated genes in the mechanisms that drive FTD progression^64,65^ and particularly TDP-43 pathogenesis^66^.

In this study, multiple innate immune and complement cascade serum proteins are dysregulated predominantly in bvFTD, some of which overlapped with ALS, indicating similar and generalized immune mechanisms are involved. Three complement component proteins representing multiple pathways of the complement cascade were shown to be dysregulated in bvFTD serum (C1S, C3, C7). Corroborating with this data, C3 was shown to be increased in bvFTD serum by western blotting in a recent study^67^. In ALS, only one complement component (C7) representing a single complement pathway was dysregulated. The complement cascade is a critical arm of innate immunity and its chronic activation is a key mediator of neuroinflammation, shown to be upregulated in the aging brain and implicated in a number of neurodegenerative diseases^68–70^, including ALS^71–73^, but largely unexplored in FTD. In one of very few studies that have addressed complement in FTD, CSF samples from FTD patients carrying the *GRN* mutation showed a progressive increase in C1q and C3 that correlated with cognitive decline^74^. Given this, and the dysregulation of a number of innate immune proteins primarily within the serum in bvFTD shown in this study, the immune/inflammatory profile in bvFTD may differ from that of ALS. Indeed, FTD and ALS have been shown previously to be characterized by a different neuroinflammatory profile using ELISA analysis of targeted inflammatory factors in CSF and blood from asymptomatic mutation carriers^75^. Collectively, these findings suggest the potential to utilize a panel of innate immune and complement serum proteins as targets for future development of disease-specific neuroinflammation in FTD and ALS. Furthermore, in the present study the dysregulated serum proteins involved in calcium binding pathways (C1S, GSN, PROS1, S100A8) overlapped with innate immunity and complement cascade pathways, suggesting a potential interplay between these two pathways and their mechanisms in bvFTD pathogenesis. Whilst distinct mechanisms in their own right, calcium and neuroinflammatory signaling pathways exhibit extensive crosstalk and bi-directional interactions^76^. Alterations in neuroinflammation with aging and disease are proposed to have strong links to dysregulated calcium signaling in glial cells of the CNS, given both microglia and astrocytes express highly developed calcium signaling machinery^77,78^.

In summary, we have demonstrated that there are significant peripheral protein changes in bvFTD and ALS serum compared to controls. In particular, proteins relating to calcium ion binding and innate immunity are dysregulated in bvFTD and ALS serum. Future work will involve further validation of these proteins in different and larger independent cohorts, and investigating their role in calcium signaling in the pathogenesis of FTD and ALS, as well as furthering the study of innate immunity. This study represents the first proteomics analysis of sporadic FTD and sporadic ALS serum together, providing new insights into under-recognized perturbed peripheral pathways and the potential for biomarker development for both FTD and ALS.

## Supplementary information

Supplementary Figure 1.

## References

[1] Piguet O, Hornberger M, Mioshi E, Hodges JR (2011). Behavioural-variant frontotemporal dementia: diagnosis, clinical staging, and management. Lancet Neurol..

[2] Kiernan MC (2011). Amyotrophic lateral sclerosis. Lancet.

[3] Westeneng HJ (2018). Prognosis for patients with amyotrophic lateral sclerosis: development and validation of a personalised prediction model. Lancet Neurol..

[4] Burrell JR (2016). The frontotemporal dementia-motor neuron disease continuum. Lancet.

[5] Neumann M (2013). Frontotemporal lobar degeneration and amyotrophic lateral sclerosis: molecular similarities and differences. Rev. Neurol. (Paris).

[6] Blair IP (2010). FUS mutations in amyotrophic lateral sclerosis: clinical, pathological, neurophysiological and genetic analysis. J. Neurol. Neurosurg. Psychiatry.

[7] Williams KL (2013). Pathophysiological insights into ALS with C9ORF72 expansions. J. Neurol. Neurosurg. Psychiatry.

[8] Feneberg E, Gray E, Ansorge O, Talbot K, Turner MR (2018). Towards a TDP-43-based biomarker for ALS and FTLD. Mol. Neurobiol..

[9] Foiani MS (2019). Searching for novel cerebrospinal fluid biomarkers of tau pathology in frontotemporal dementia: an elusive quest. J. Neurol. Neurosurg. Psychiatry.

[10] Zetterstrom P, Andersen PM, Brannstrom T, Marklund SL (2011). Misfolded superoxide dismutase-1 in CSF from amyotrophic lateral sclerosis patients. J. Neurochem..

[11] Rohrer JD (2016). Serum neurofilament light chain protein is a measure of disease intensity in frontotemporal dementia. Neurology.

[12] Verde F (2019). Neurofilament light chain in serum for the diagnosis of amyotrophic lateral sclerosis. J. Neurol. Neurosurg. Psychiatry.

[13] Pandey A, Mann M (2000). Proteomics to study genes and genomes. Nature.

[14] Dey KK (2019). Deep undepleted human serum proteome profiling toward biomarker discovery for Alzheimer's disease. Clin. Proteomics.

[15] Xu Z, Lee A, Nouwens A, Henderson RD, McCombe PA (2018). Mass spectrometry analysis of plasma from amyotrophic lateral sclerosis and control subjects. Amyotroph. Lateral Scler. Frontotemporal Degener..

[16] Bereman MS, Beri J, Enders JR, Nash T (2018). Machine learning reveals protein signatures in CSF and plasma fluids of clinical value for ALS. Sci. Rep..

[17] Zubiri I (2018). Tissue-enhanced plasma proteomic analysis for disease stratification in amyotrophic lateral sclerosis. Mol. Neurodegener..

[18] Ahmed RM (2014). Systemic metabolism in frontotemporal dementia. Neurology.

[19] Rascovsky K (2011). Sensitivity of revised diagnostic criteria for the behavioural variant of frontotemporal dementia. Brain.

[20] Al-Chalabi A (2016). Amyotrophic lateral sclerosis: moving towards a new classification system. Lancet Neurol..

[21] Zolotarjova N, Mrozinski P, Chen H, Martosella J (2008). Combination of affinity depletion of abundant proteins and reversed-phase fractionation in proteomic analysis of human plasma/serum. J. Chromatogr. A.

[22] Ting L, Rad R, Gygi SP, Haas W (2011). MS3 eliminates ratio distortion in isobaric multiplexed quantitative proteomics. Nat. Methods.

[23] Cox J, Mann M (2008). MaxQuant enables high peptide identification rates, individualized p.p.b.-range mass accuracies and proteome-wide protein quantification. Nat. Biotechnol..

[24] Antonov AV (2011). BioProfiling.de: analytical web portal for high-throughput cell biology. Nucleic Acids Res..

[25] Szklarczyk D (2019). STRING v11: protein–protein association networks with increased coverage, supporting functional discovery in genome-wide experimental datasets. Nucleic Acids Res..

[26] Molania R, Gagnon-Bartsch JA, Dobrovic A, Speed TP (2019). A new normalization for Nanostring nCounter gene expression data. Nucleic Acids Res..

[27] Ritchie ME (2015). limma powers differential expression analyses for RNA-sequencing and microarray studies. Nucleic Acids Res..

[28] Davidsson P (2002). Studies of the pathophysiological mechanisms in frontotemporal dementia by proteome analysis of CSF proteins. Brain Res. Mol. Brain Res..

[29] Teunissen CE (2016). Novel diagnostic cerebrospinal fluid biomarkers for pathologic subtypes of frontotemporal dementia identified by proteomics. Alzheimers Dement. (Amst).

[30] van der Ende EL (2019). Novel CSF biomarkers in genetic frontotemporal dementia identified by proteomics. Ann. Clin. Transl. Neurol..

[31] Barschke P, Oeckl P, Steinacker P, Ludolph A, Otto M (2017). Proteomic studies in the discovery of cerebrospinal fluid biomarkers for amyotrophic lateral sclerosis. Expert Rev. Proteomics.

[32] Sudhof TC (2012). Calcium control of neurotransmitter release. Cold Spring Harb. Perspect. Biol..

[33] Emptage NJ, Reid CA, Fine A (2001). Calcium stores in hippocampal synaptic boutons mediate short-term plasticity, store-operated Ca^2+^ entry, and spontaneous transmitter release. Neuron.

[34] Lu B (2010). Extracellular calcium controls background current and neuronal excitability via an UNC79–UNC80–NALCN cation channel complex. Neuron.

[35] Grosskreutz J, Van Den Bosch L, Keller BU (2010). Calcium dysregulation in amyotrophic lateral sclerosis. Cell Calcium.

[36] Palluzzi F (2017). A novel network analysis approach reveals DNA damage, oxidative stress and calcium/cAMP homeostasis-associated biomarkers in frontotemporal dementia. PLoS ONE.

[37] Beltran-Parrazal L, Charles A (2003). Riluzole inhibits spontaneous Ca^2+^ signaling in neuroendocrine cells by activation of K^+^ channels and inhibition of Na^+^ channels. Br. J. Pharmacol..

[38] Imamura K (2016). Calcium dysregulation contributes to neurodegeneration in FTLD patient iPSC-derived neurons. Sci. Rep..

[39] Bursch F (2019). Altered calcium dynamics and glutamate receptor properties in iPSC-derived motor neurons from ALS patients with C9orf72, FUS, SOD1 or TDP43 mutations. Hum. Mol. Genet..

[40] Dafinca R (2016). C9orf72 hexanucleotide expansions are associated with altered endoplasmic reticulum calcium homeostasis and stress granule formation in induced pluripotent stem cell-derived neurons from patients with amyotrophic lateral sclerosis and frontotemporal dementia. Stem Cells.

[41] Aggad D, Veriepe J, Tauffenberger A, Parker JA (2014). TDP-43 toxicity proceeds via calcium dysregulation and necrosis in aging *Caenorhabditis**elegans* motor neurons. J. Neurosci..

[42] Feldt J (2019). Structure, regulation and related diseases of the actin-binding protein gelsolin. Expert Rev. Mol. Med..

[43] Piktel E, Levental I, Durnas B, Janmey PA, Bucki R (2018). Plasma gelsolin: indicator of inflammation and its potential as a diagnostic tool and therapeutic target. Int. J. Mol. Sci..

[44] Choe H (2002). The calcium activation of gelsolin: insights from the 3A structure of the G4–G6/actin complex. J. Mol. Biol..

[45] Dahlback B (1991). Protein S and C4b-binding protein: components involved in the regulation of the protein C anticoagulant system. Thromb. Haemost..

[46] Tondo G, Perani D, Comi C (2019). TAM receptor pathways at the crossroads of neuroinflammation and neurodegeneration. Dis Mark..

[47] Ohsawa I, Takamura C, Kohsaka S (2001). Fibulin-1 binds the amino-terminal head of beta-amyloid precursor protein and modulates its physiological function. J. Neurochem..

[48] Zhang Y, Marmorstein LY (2010). Focus on molecules: fibulin-3 (EFEMP1). Exp. Eye Res..

[49] Xu S (2014). Role of fibulin-3 in lung cancer: in vivo and in vitro analyses. Oncol. Rep..

[50] Brancaccio P, Limongelli FM, Maffulli N (2006). Monitoring of serum enzymes in sport. Br. J. Sports Med..

[51] Palumbo B, Siepi D, Sabalich I, Tranfaglia C, Parnetti L (2008). Cerebrospinal fluid neuron-specific enolase: a further marker of Alzheimer's disease?. Funct. Neurol..

[52] Schmidt FM (2014). Elevated levels of cerebrospinal fluid neuron-specific enolase (NSE) in Alzheimer's disease. Neurosci. Lett..

[53] Alzheimer's Association Calcium Hypothesis Workgroup (2017). Calcium hypothesis of Alzheimer's disease and brain aging: a framework for integrating new evidence into a comprehensive theory of pathogenesis. Alzheimers Dement..

[54] Fairless R, Williams SK, Diem R (2019). Calcium-binding proteins as determinants of central nervous system neuronal vulnerability to disease. Int. J. Mol. Sci..

[55] Zundorf G, Reiser G (2011). Calcium dysregulation and homeostasis of neural calcium in the molecular mechanisms of neurodegenerative diseases provide multiple targets for neuroprotection. Antioxid. Redox Signal..

[56] Ising C, Heneka MT (2018). Functional and structural damage of neurons by innate immune mechanisms during neurodegeneration. Cell Death Dis..

[57] Heneka MT, Kummer MP, Latz E (2014). Innate immune activation in neurodegenerative disease. Nat. Rev. Immunol..

[58] Bright F (2019). Neuroinflammation in frontotemporal dementia. Nat. Rev. Neurol..

[59] Liu J, Wang F (2017). Role of neuroinflammation in amyotrophic lateral sclerosis: cellular mechanisms and therapeutic implications. Front. Immunol..

[60] Lant SB (2014). Patterns of microglial cell activation in frontotemporal lobar degeneration. Neuropathol. Appl. Neurobiol..

[61] Corcia P (2012). Molecular imaging of microglial activation in amyotrophic lateral sclerosis. PLoS ONE.

[62] Zhang R (2011). Gene expression profiling in peripheral blood mononuclear cells from patients with sporadic amyotrophic lateral sclerosis (sALS). J. Neuroimmunol..

[63] McCauley ME, Baloh RH (2019). Inflammation in ALS/FTD pathogenesis. Acta Neuropathol..

[64] Broce I (2018). Immune-related genetic enrichment in frontotemporal dementia: an analysis of genome-wide association studies. PLoS Med..

[65] Ferrari R (2014). Frontotemporal dementia and its subtypes: a genome-wide association study. Lancet Neurol..

[66] Pottier C (2019). Genome-wide analyses as part of the international FTLD-TDP whole-genome sequencing consortium reveals novel disease risk factors and increases support for immune dysfunction in FTLD. Acta Neuropathol..

[67] Phan K (2020). Uncovering pathophysiological changes in frontotemporal dementia using serum lipids. Sci. Rep..

[68] Stephan AH, Barres BA, Stevens B (2012). The complement system: an unexpected role in synaptic pruning during development and disease. Annu. Rev. Neurosci..

[69] Stephan AH (2013). A dramatic increase of C1q protein in the CNS during normal aging. J. Neurosci..

[70] Hammond TR, Marsh SE, Stevens B (2019). Immune signaling in neurodegeneration. Immunity.

[71] Mantovani S (2014). Elevation of the terminal complement activation products C5a and C5b–9 in ALS patient blood. J. Neuroimmunol..

[72] Woodruff TM, Lee JD, Noakes PG (2014). Role for terminal complement activation in amyotrophic lateral sclerosis disease progression. Proc. Natl. Acad. Sci. U.S.A..

[73] Ganesalingam J (2011). Combination of neurofilament heavy chain and complement C3 as CSF biomarkers for ALS. J. Neurochem..

[74] Lui H (2016). Progranulin deficiency promotes circuit-specific synaptic pruning by microglia via complement activation. Cell.

[75] Oeckl P (2019). Different neuroinflammatory profile in amyotrophic lateral sclerosis and frontotemporal dementia is linked to the clinical phase. J. Neurol. Neurosurg. Psychiatry.

[76] Sama DM, Norris CM (2013). Calcium dysregulation and neuroinflammation: discrete and integrated mechanisms for age-related synaptic dysfunction. Ageing Res. Rev..

[77] Farber K, Kettenmann H (2006). Functional role of calcium signals for microglial function. Glia.

[78] Verkhratsky A, Rodriguez JJ, Parpura V (2012). Calcium signalling in astroglia. Mol. Cell. Endocrinol..

