# Supplementary material for: Altered serum protein levels in frontotemporal dementia and amyotrophic lateral sclerosis indicate calcium and immunity dysregulation
Source: Sci Rep. 2020 Aug 13;10:13741. doi: 10.1038/s41598-020-70687-7 (PMC7426269; doi:10.1038/s41598-020-70687-7)
Supplement: Supplementary file 1 — Supplementary Figure 1. [file 41598_2020_70687_MOESM1_ESM.docx]

Supplementary Information

**Altered serum protein levels in frontotemporal dementia and amyotrophic lateral sclerosis indicate calcium and immunity dysregulation**

Jared S. Katzeff^1^, Fiona Bright^2^, Kitty Lo^3^, Jillian J. Kril^2^, Angela Connolly^4^, Ben Crossett^4^, Lars M. Ittner^5^, Michael Kassiou^6^, Clement T. Loy^1,7^, John R. Hodges^1^, Olivier Piguet^1,8,9^, Matthew C. Kiernan^1,10^, Glenda M. Halliday^1,2,9,11*^ and Woojin Scott Kim^1,9,11*^

**Supplementary Figure 1**

Validation of proteomics data by western blotting. The full-size blots of the cropped images as shown in Figure 5. (A) COMP. (B) GSN. (C) Transferrin. (D) EFEMP1. (E) PROS1. (F) Transferrin. (G) FBLN1. (H) Transferrin. (I) COMP. (J) GSN. (K) Transferrin. (L) CKM. (M) Transferrin.

100kDa

90kDa

75kDa

55kDa

70kDa

75kDa

100kDa

75kDa

100kDa

90kDa

75kDa

45kDa

75kDa
